# A Real-Time Vision-Based Adaptive Follow Treadmill for Animal Gait Analysis

**DOI:** 10.3390/s25144289

**Published:** 2025-07-09

**Authors:** Guanghui Li, Salif Komi, Jakob Fleng Sorensen, Rune W. Berg

**Affiliations:** Department of Neuroscience, Faculty of Health and Medical Sciences, University of Copenhagen, DK-2200 Copenhagen N, Denmark; salif.komi@sund.ku.dk (S.K.); jakob.fleng@sund.ku.dk (J.F.S.)

**Keywords:** real-time computer vision, intelligent treadmill, adaptive control, OpenMV4, object tracking, FOMO MobileNetV2

## Abstract

Treadmills are a convenient tool to study animal gait and behavior. Traditional animal treadmill designs often entail preset speeds and therefore have reduced adaptability to animals’ dynamic behavior, thus restricting the experimental scope. Fortunately, advancements in computer vision and automation allow circumvention of these limitations. Here, we introduce a series of real-time adaptive treadmill systems utilizing both marker-based visual fiducial systems (colored blocks or AprilTags) and marker-free (pre-trained models) tracking methods powered by advanced computer vision to track experimental animals. We demonstrate their real-time object recognition capabilities in specific tasks by conducting practical tests and highlight the performance of the marker-free method using an object detection machine learning algorithm (FOMO MobileNetV2 network), which shows high robustness and accuracy in detecting a moving rat compared to the marker-based method. The combination of this computer vision system together with treadmill control overcome the issues of traditional treadmills by enabling the adjustment of belt speed and direction based on animal movement.

## 1. Introduction

Treadmills are commonly used for investigating rodent gait and behavior, with extensive applications in neuroscience, rehabilitation medicine, and exercise biology [1,2,3,4,5,6,7,8,9,10]. Traditional treadmill designs are relatively simplistic, requiring researchers to preset parameters for speed and direction before and during the experiment. Hence, the animal is forced to follow these settings, which limit the scope of the investigation of autonomous movement intentions and coordination abilities. This is especially noted for injury-induced models, where sustaining stable and continuous movement is challenging. Animals frequently require immobilization using specialized support structures to achieve stable gait monitoring during exercise [11,12], leading to completely involuntary movement. Moreover, a more accommodating treadmill will improve the investigation of the spinal motor networks during movement dynamics [13], which are influenced by both the neural control mechanisms and the external physical environment. These deficiencies constrain the assessment of contents, such as movement intent, motor planning, and control capabilities. Therefore, there is a demand for a novel treadmill design that can adapt to changes with animal movement speed and directions by adjusting the treadmill motor speed in real-time.

With the rapid advancement of automation and computer vision powered by deep learning [14,15,16], alongside the continuous emergence of microelectronic products, these developments offer convenient improvements in the experimental design [17,18,19,20,21,22,23,24].

In this paper, we use an embedded vision platform (OpenMV) that can significantly reduce system design complexity and further ensure the stability of the system. The latest OpenMV4 Plus version model can run multiple algorithms for target detection and recognition tasks, including face detection, QR code recognition, and barcode recognition, among other targets. Spence et al. introduced a treadmill-based tracking system that uses Kalman filters to analyze video data, providing real-time estimations of locomotor velocity and position on the treadmill [25]. As an optional solution, we provided a marker-based (colored blocks or AprilTags) tracking method that requires a simpler machine vision algorithm and has a high real-time performance. A red color block or AprilTag was attached to the sides of animal’s body as the recognition target; the animal’s position is detected by tracking the center coordinate of the marker.

The stability of tracking results can be affected by multiple factors such as the texture of the animal’s fur, the lighting environment, and the deformation caused by motion. Those issues can be further addressed by introducing deep learning techniques into the object detection tasks, which have made significant progress and shown excellent performance in image processing [26,27,28,29,30,31]. However, the device limitations and memory resources restrict their ability to run complex deep-learning models. It is necessary to run simplified or optimized models to achieve a higher recognition accuracy. Beyond that, when the real-time performance and low-power requirements need to be taken into consideration, there is usually a trade-off between performance and functionality. 

In another aspect, we introduced the marker-free method based on a deep learning model, which was trained with an object detection machine learning algorithm, the FOMO MobileV2 network, as a convenient solution to eliminate the need for physical markers (like color blocks or AprilTags). Instead, it relies on the natural features of the animal for real-time object detection and tracking. The training performance of this neural network demonstrates excellent classification ability in dynamic environments where the animal is constantly changing position and posture on the treadmill.

## 2. Materials and Methods

### 2.1. Setup and Working Principles

#### 2.1.1. Mechanical and Electrical Setup

**Figure 1:**
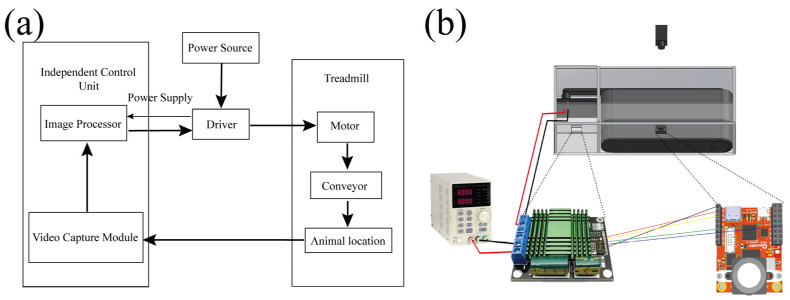
Block diagram of the system design (**a**), and the circuit diagram between the hardware components (**b**).

Figure 1 illustrates the basic principle of the system design. Based on the OpenMV machine vision module, the independent control unit functions to provide image acquisition and processing as well as motor control. The drive module receives power from the power source and provides current required for driving the motor (3XE22, Dayton), and it further drives the mechanical parts of treadmill to work. This forms a closed-loop control system, enabling the system to be controlled automatically (Figure 1a). The hardware components of the system consist of a power source (MFG KA6003D, SRA Soldering Products, Walpole, MA, USA), an independent control unit (Openmv4), a driver module (meovzzy0x3xw121, 60A High Power MOS Dual Channel H-Bridge DC Motor Driver Module Xt90), and the mechanical parts of treadmill (Figure 1b).

The mechanical part of the treadmill consists of the drive motor, the conveyor wheels, and the support frame. In our design, the motor and mechanical parts are taken from a commercial animal platform (TREADMILL SIMPLEX II: Exer3/6 COLUMBUS INSTRUMENT). The control panel was removed, leaving the motor and conveyor wheels as well as the support frame. Then, the power supply, controller, and drives were added and integrated into a new automation system. The design shows a universal adaptability, allowing researches to modify their existing treadmill for intelligent applications.

#### 2.1.2. Vision-Based Animal Tracking 

The OpenMV camera module is used as a motor driver, and it features open-source, cost-effective, and notably potent machine vision support. It is powered by the STM32F765 ARM microcontroller and integrates OmniVsion’s OV7725 VGA CMOS sensor and the L298 motor drive module. OpenMV uses Python 3.0 programming language with embedded image sensor and advanced machine vision features, functioning as a programmable camera module [32].

**Figure 2:**
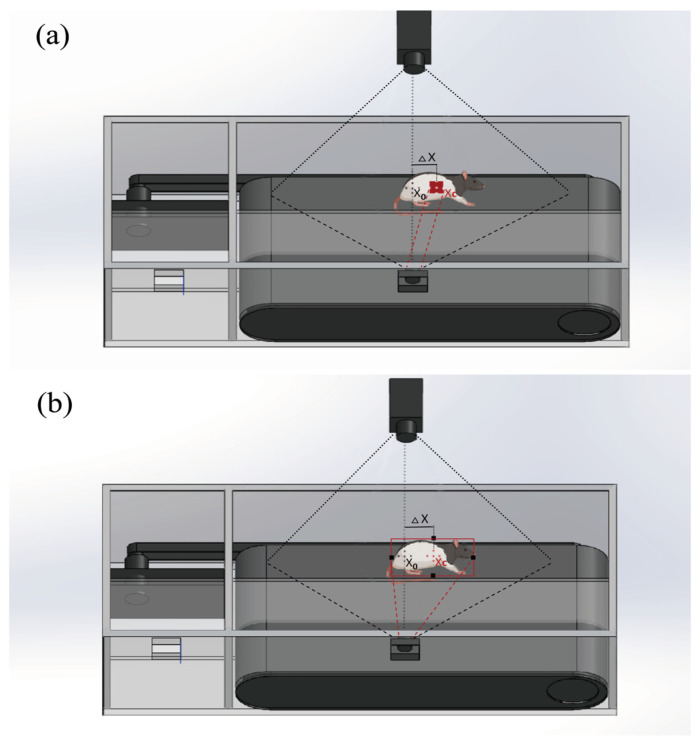
Real-time vision-based control methods that employ color block- or AprilTag-based tracking methods (**a**), and the trained visual tracking model-based method to detect and monitor objects (**b**).

The OV7725 VGA CMOS sensor acquires images and subsequently employs built-in image processing algorithms. This enables the system to compute the coordinates of the central point of target images and the field of view, which is commonly used in the field of computer vision and imaging processing. The deviation is obtained by subtracting those two coordinates. Then, the result is used for controlling motor speed and direction (Figure 2). Namely, the motor’s rotational direction depends on whether it is positive or negative, and speed is linearly correlated with the absolute value of the deviation. The frame structure of the treadmill is constructed with transparent acrylic material, with the behavioral detection camera oriented vertically toward the animal’s location.

By deploying OpenMV as an artificial vision system for position detection, it analyses the information from the camera and controls the motor’s direction and speed. The motor’s speed and direction are dynamically adjusted based on the X-axis distance of the center position between the color block and the OpenMV camera’s field of view, ensuring that the animal remains stably positioned on the treadmill. The animal’s position is always focused on the center of the behavioral camera’s field of view, facilitating the sustained and stable capture of images for analysis of the animal’s gait and behavior. The detailed control method is described as follows.

The center coordinates of the field of view are calculated as follows:

(1) $$x_{0}=\frac{W}{2},\;y_{0}=\frac{H}{2}$$

where *W* and *H* are the width and height of the image captured by the OpenMV Cam camera, respectively.

The center coordinates of the target object are calculated as follows:

(2) $$x_{c}=\frac{x_{min}+x_{max}}{2},\;y_{c}=\frac{y_{min}+y_{max}}{2}$$

Here, $\left(x_{min},y_{min}\right)$ is the upper left corner of the target bounding box, $\left(x_{max},y_{max}\right)$ is the lower right corner of the target object bounding box, and $\left(x_{c},y_{c}\right)$ represents the coordinates of the target center of the detected object. In the pre-trained model-based detection tasks, the center coordinates can be output directly by the heatmap layer of the FOMO network architecture.

Then, we obtain the deviation value between the center of mass of the target object and the center of the image data as follows:

(3) $$\Delta x=x_{c}-x_{0},\;\Delta y=y_{c}-y_{0}$$

where *Dx* and *Dy* are the deviation values in the horizontal and vertical directions, respectively.

#### 2.1.3. Treadmill Control Algorithm and Working Principle

The adopted algorithms for motor direction control and speed adjustment are relatively simple. The motor’s rotational direction is opposite to the sign of the relative displacement. Therefore, when the object is in motion, the motor will rotate in the opposite direction. The motor’s rotational speed has a linear relationship with the absolute value of the displacement, and the speed increases as the absolute value of the displacement increases. The classical proportional–integral–derivative (PID) control method was used to dynamically adjust values for the speed and direction of the treadmill based on the deviation value as follows [33]:

(4) $$u_{adjust}\left(t\right)=K_{p}\cdot \Delta x+K_{i}\cdot \int \Delta xdt+K_{d}\cdot \frac{d\left(\Delta x\right)}{dt}$$

Here,$\;u_{adjus}\left(t\right)$ is the adjustment amount of the treadmill; and $K_{p},K_{i},andK_{d}$ represent the proportional gain, integral gain, and differential gain, respectively.

The adjustment amount determines the movement direction and speed of the treadmill and is converted into control signal to adjust the moving object to the preset position of the treadmill as follows:

(5) $$v_{adjust}=k\cdot \left|u_{adjust}\left(t\right)\right|$$

where $u_{adjust}$ is the adjusted speed of the treadmill motor, and k is the coefficient proportional to speed.

Due to the different resistances of the belts of treadmills, in practice, the user needs to adjust the PID parameters and test the smoothness of the motor’s rotation.

**Figure 3:**
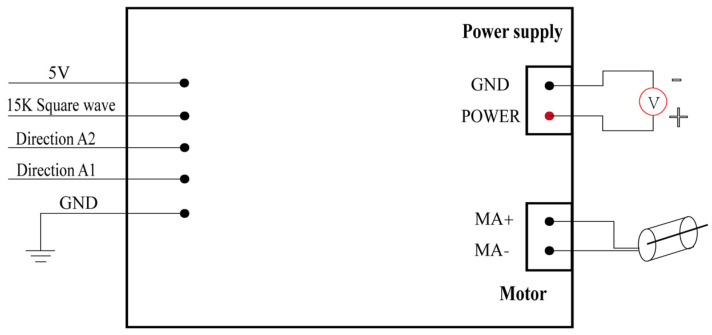
An H−bridge DC motor driver model links the motor correlative control signal and upper controller.

We utilized an H-bridge DC motor driver module with high power output capacity to drive the motor’s rotation direction and speed. The control principle for the DC motor is as follows: A1.A2 = 0.0 for the brake; A1.A2 = 1.0 for forward rotation; A1.A2 = 0.1 for reverse rotation. PWM is used as the input signal to drive the motor under the control of a 15 K Hz square wave for motor speed regulation A 5 V supply power is provided to the OpenMV module, with a common ground connection for the driver board and the OpenMV control module (Figure 3).

#### 2.1.4. Control Program

The system control program is divided into three parts, labeled as ‘motor’, ‘pid’ and ‘main’, separately. Among them, ‘motor’ is used to control the motor’s forward and reverse rotation as well as speed control. The ‘pid’ part is used for PID control of the PWM signal. The ‘main’ part serves as the main program that calls ‘motor’ and ‘pid’, which is used for object recognition, extraction of pixel center positions, and calculation of center point offset displacement.

**Figure 4:**
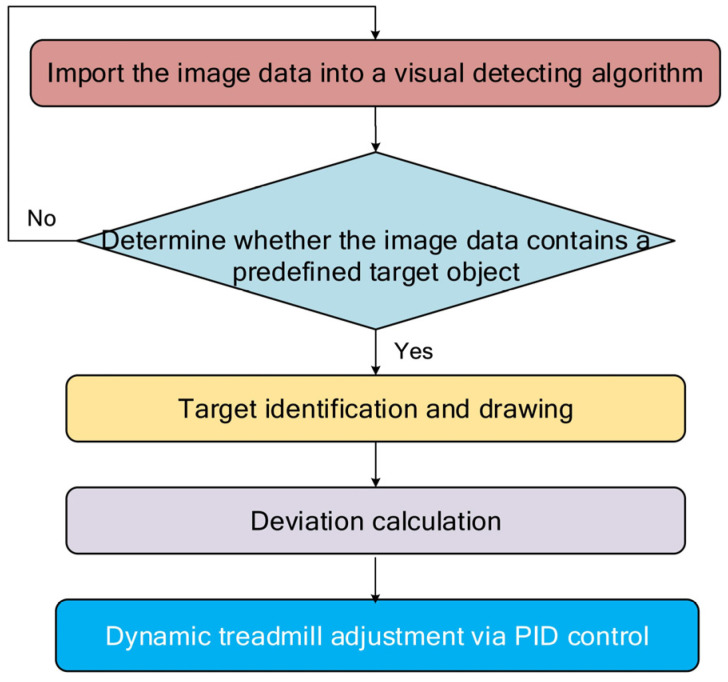
A flow chart describing the functioning of the Python 3.0 code in the ‘main’ part.

The system uses a consistent framework for detecting different types of targets, such as colored markers, AprilTags, or pre-trained models. For different types of target detection, similar methods are employed with the specific algorithm adjusted depending on the type of target. Figure 4 illustrates the general approach. The image processing workflow for the intelligent treadmill begins by importing real-time video frames into a visual tracking algorithm to detect the presence of the target object—such as a color marker, AprilTag, or unmarked animal body. For marker-based tracking (e.g., red blocks in Supplemental Videos 1 and 2 provided with source code in our public repository (https://github.com/ghli001/Real-Time-Vision-Based-Adaptive-Follow-Intelligent-Treadmill (accessed on 1 July 2025))), the system applies predefined color and area thresholds to identify the largest valid target and filters out noise. Once a target is detected, the system visualizes key features, including its bounding box, major and minor axes, and centroid. It then calculates the deviation between the target’s centroid and the center of the image frame. Using this deviation, the treadmill’s motion is dynamically adjusted using a PID controller to maintain the subject at a predefined central position on the treadmill belt, ensuring continuous, adaptive alignment for behavioral tracking.

### 2.2. Marker-Free Adaptive Follow 

#### 2.2.1. Model Architecture Overview

To track rats directly from their body features, we adopted the FOMO MobileNetV2 network for the training of our marker-free object detection model. FOMO (Fast Object Detection and Tracking) uses MobileNetV2 as a backbone for its lightweight object detection of mobile devices and resource-constrained environments [34]. MobileNetV2 itself is known for its efficiency, utilizing depth-wise separable convolutions and inverse residuals to minimize computational costs and memory usage without sacrificing performance [35,36]. These features make it ideal for applications in resource-limited environments. FOMO MobileNetV2 takes these principles further by removing the fully connected layers and the final convolutional layers, turning the network’s output into a compact heatmap that directly encodes the location and class probability of objects. This architectural change allows FOMO to achieve high performance in object detection tasks with minimal computational requirements [34]. Compared with standard MobileNetV2, it is more compact and flexible, is very fast in object detection tasks, and provides detailed information on the precious location, size, and quantity of the target object. This is crucial for object detection and tracking tasks, making it a valuable solution for real-time single-target detection and embedded device applications.

**Figure 5:**
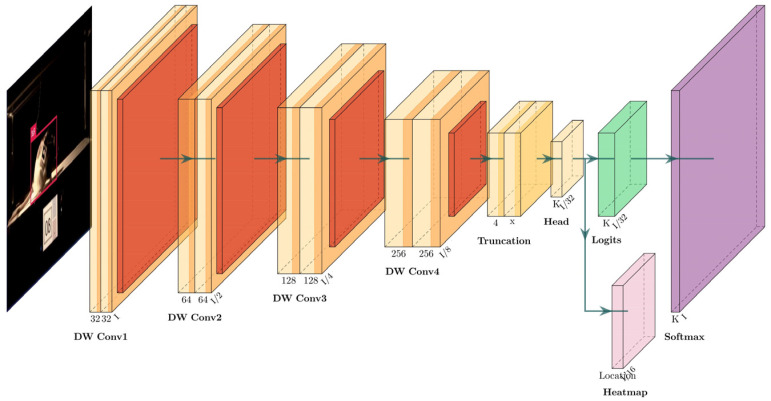
The architecture of FOMO MobileNetV2 network, including the original image as the input layer, MobileNetV2 convolutional blocks (DW Conv1, DW Conv2, DW Conv3, DW Conv4), truncation layer, FOMO-specific layers (head layer, logits layer, softmax layer), and heatmap layer.

Figure 5 represents a diagram generated by PlotNeuralNet [37] to visualize the FOMO MobileNetV2 architecture, reflecting how the data flows from the input through various convolutional and detection-specific layers, culminating in a heatmap for object localization. The input size is 160 × 160 × 3, which is placed at the leftmost part of the diagram. The network includes four depth-wise separable convolutional blocks (DW Conv1, DW Conv2, DW Conv3, DW Conv4) with increasing channel sizes (32, 64, 128, 256). These blocks are represented with custom colors for convolution and ReLU activation. Followed by the truncation layer, FOMO utilizes MobileNetV2 as a feature extractor but truncates the network after a specific layer to generate a reduced feature map with dimensions 4 × 4 × n, where “n” corresponds to the number of object classes in the dataset. This truncation enables the model to focus on relevant features for object detection rather than classification alone. The subsequent layers in FOMO consist of the head layer, which performs feature extraction specific to object detection, and the logits layer, which generates raw predictions for each class. These logits are then passed through a Softmax layer to generate class probabilities. The heatmap layer is unique to FOMO MobileNetV2 and is used to generate heatmaps for object localization instead of bounding boxes for object detection.

#### 2.2.2. Model Training and Deployment

Long Evans rats were used as testing subjects. Each rat had a uniquely trained model to minimize the influence of variable environmental and body feature factors on model performance. We standardized all pertinent environmental factors, including illumination, camera angle, and field of view, to ensure consistency between the training and real-world environments. For models deployed within a polycarbonate enclosure, testing indicated that black backgrounds yielded optimal performance; consequently, a black background was selected for these conditions.

The model was trained using Edge Impulse, a platform optimized for machine learning models tailored for embedded systems and edge devices. The full project is publicly available at https://studio.edgeimpulse.com/public/514241/live (accessed on 1 July 2025). The training dataset consisted of 207 annotated images, and 42 annotated images were reserved for testing. Bounding boxes were used to annotate and calibrate the position of the rats. We selected FOMO MobileNetV2 0.35 as the model architecture due to its larger width multiplier compared to FOMO MobileNetV2 0.1, offering improved accuracy while maintaining computational efficiency. The model was trained for 60 epochs using the Adam optimizer with a learning rate of 0.001. The objectness-weighted focal loss was employed to improve performance in anchor-free detection tasks. Standard data augmentation strategies included random horizontal flips, brightness and contrast adjustments, mild rotations (±10°), and input rescaling. The performance of the model was evaluated using the F1 score, a metric that balances precision and recall. A model exceeding 95% will be used for deployment. An error classification analysis was conducted to assess the impact of misclassifications on task performance, ensuring the model’s reliability in practical applications. During runtime, the system achieved an inference time of approximately 87 ms per frame (~11.5 FPS), with peak RAM usage of 239 KB and flash usage of 79.0 KB. These results were measured using Edge Impulse’s on-device profiler, and we further modified the control script to log clock.fps() regularly, providing real-time feedback on system throughput and latency. After training, the model was integrated into executing scripts that combined object recognition with motion control. The scripts were packaged into a .zip file and deployed to the OpenMV4 Cam H7 Plus.

## 3. Results and Discussion

By evaluating the training process and the classification performance, the neural network demonstrated excellent performance by the end of the training. It effectively minimizes both training and validation loss while achieving high precision, recall, and F1 scores. The steady alignment of training and validation metrics suggests the model generalizes well without overfitting, making it a robust and reliable model for the marker-free object detecting task.

**Figure 6:**
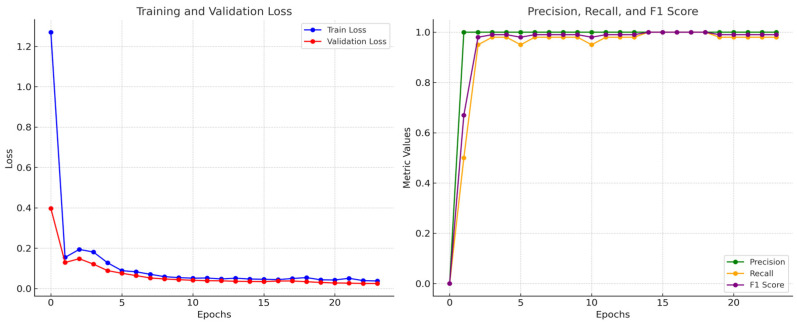
Training steps and performance of the model.

Figure 6 provides insights into the neural network’s training process, showing that the model achieved good performance with minimal overfitting. The training proceeded for 60 epochs in total, and the loss decreases consistently across epochs, starting at 1.27027 in epoch 0, falling to 0.03321 by epoch 24, and stabilizing afterward. The sharp decline in training loss indicates that the model is learning effectively and fitting the training data well. The validation loss also decreases steadily, starting at 0.39714 in epoch 0 and reducing to 0.02274 by epoch 24, with minimal fluctuations afterward. The validation loss closely follows the training loss, which indicates the model is generalizing well to unseen data and is not overfitting to the training data. The model’s fast convergence is likely due to the controlled environment of the treadmill setting, where variations in lighting, background, and object morphology are limited. Such conditions simplify the learning problem and reduce overfitting risk.

Meanwhile, the training performance of this neural network, based on the provided precision, recall, and F1 score values, indicates the model achieved excellent performance with near-perfect metrics. The training performance of this neural network demonstrated remarkable classification ability. With a precision of 1.00, the model indicates that there were no false positives after epoch 1. The recall ranges from 0.98 to 1.00, demonstrating the model’s capability to identify nearly all relevant instances by epoch 14, and an F1 score between 0.99 and 1.00 reflects an almost perfect balance between precision and recall. These results suggest that the model has effectively learned the task and generalized well to the validation data.

**Figure 7:**
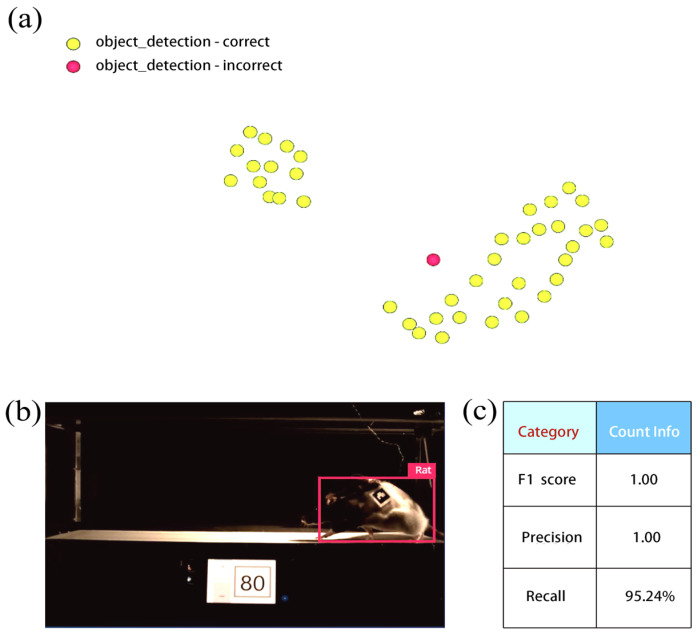
The model testing output, which shows all data in the test set classified by the neural network. Items in green are classified correctly, and items in red are misclassified (**a**). Deep insight into the misclassified case (**b**) and metrics for object detection using this model (**c**).

After training, we analyzed the classification performance using validation results from both the full-precision (float32) and quantized (int8) models. The float32 model achieved perfect classification (100% precision, recall, and F1 score) for both classes. In contrast, the quantized int8 model exhibited slightly lower recall (95.24%), reflecting a minor misclassification, while maintaining perfect precision and a high F1 score (97.6%) for the “Rat” class (Table 1). This modest trade-off is characteristic of quantized models deployed on edge devices, where gains in computational efficiency may come at the expense of marginal reductions in recall. Despite this, the model demonstrates robust detection performance under controlled treadmill conditions. Nonetheless, the model continues to demonstrate strong performance in detecting rats accurately in controlled treadmill conditions. To further examine this behavior, we analyzed the misclassified instance (Figure 7a) using FOMO and found that the model successfully detected and labeled the rat, but its prediction collapsed to the object’s centroid (Figure 7b).

This may be attributed to FOMO’s design, which prioritizes centroids for efficient object detection rather than complete bounding boxes. When the object spans multiple grid cells but the network is overly focused on predicting centroids, it may “collapse” the detection to a single grid cell (the centroid), even when the object is recognized accurately. This issue can further be mitigated by increasing grid resolution to better represent objects that span multiple cells and adjusting the loss function to balance centroid predictions with spatial consistency across the object’s extent. This ensures that the training data include diverse object sizes and positions to teach the model to predict beyond just centroids. However, this issue reflects a trade-off in lightweight models like FOMO, where efficiency and simplicity may occasionally come at the cost of spatial precision. The detailed parameters of the model’s performance for object detection (including accuracy, precision, and F1 score) are summarized in Figure 7c. These parameters reflect its near-perfect object detection ability.

We evaluated the performance of this model in real-world tasks. In practical applications, the movement of animals involves changes in gait and dynamic adjustments of body posture and will cause significant deformation; thus, tracking errors caused by deformation are difficult to avoid.

**Figure 8:**
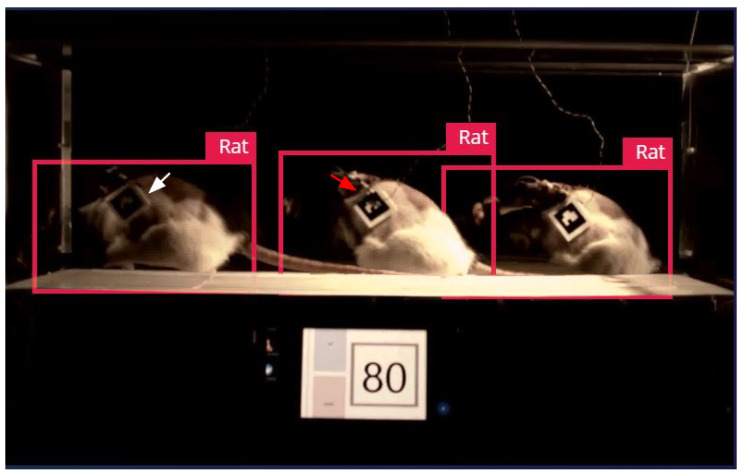
Evaluating the performance of the marker-free method in real-world task where detection failed using AprilTags (labeled with white and red arrows).

In several cases where label detection failed due to environmental lighting (indicated as white arrow in Figure 8) and deformation (shown as red arrow in Figure 8) when using AprilTags as recognition targets, our pre-trained model-based visual recognition method detected the rat’s morphology successfully and demonstrated almost perfect accuracy and robustness.

## 4. Limitations

In this paper, we propose a series of intelligent upgrades for animal treadmills by leveraging our existing mechanical structures. These upgrades are compatible with traditional gait research paradigms. Notably, recent advancements in human rehabilitation have introduced an omnidirectional treadmill that allows for dynamic changes in speed and direction guided by virtual reality, facilitating more complex locomotor tasks [38]. This innovation offers valuable insights for traditional behavioral studies [39,40,41]. Developing similar devices tailored for experimental animals could potentially broaden the scope of conventional research methodologies.

We used OpenMV Cam as an embedded vision platform and one H-bridge driver board to achieve target recognition and positioning. It is a simple and cost-effective solution to modify our existing laboratory treadmill with a total cost of less than USD 150. It is important to note that the overall design of this intelligent treadmill system uses DC motors. Due to the significant electromagnetic noise generated by DC motors, in experiments requiring electrophysiological data collection, there might be substantial noise interference. Therefore, replacing it with a brushless servo motor would be a better choice for such experiments.

In our study, the PID parameters were empirically tuned using iterative testing in a controlled environment. The tuning process aimed to balance responsiveness (reducing latency in speed adaptation) with smoothness (avoiding oscillations that could affect animal behavior). In future work, we plan to implement adaptive or model-based control methods and quantitatively evaluate PID tuning under varying gait dynamics and environmental conditions.

Finally, latency remains an inherent limitation in embedded systems, particularly under constrained computational resources. As emphasized throughout this work, our primary goal is to provide a cost-effective, open, and modular platform that can be adopted by the research community to retrofit traditional treadmills with modern control capabilities using off-the-shelf embedded components. Despite hardware limitations, the current implementation demonstrates viable real-time performance, with inference latency measured at approximately 87 ms per frame. Nonetheless, future deployment on more powerful embedded platforms—such as Cortex-M7, Cortex-A, ARC, or ESP32-S3—could help further reduce latency and enhance the responsiveness of the system in more demanding experimental conditions.

## 5. Conclusions

In this study, we provided adaptive treadmills and control methods based on real-time computer vision and demonstrated the capabilities of the FOMO MobileNetV2 model for real-time object detection in resource-constrained environments. We ensured the reliability of the model in real-world applications by integrating the model into OpenMV4 Cam H7 Plus and optimizing the model so that it can run efficiently on embedded systems while maintaining high accuracy.

## Figures and Tables

**Table 1:** Classification report on the test set (int8).

Class	Precision	Recall	F1 Score	Accuracy	Support
Background (*class 0*)	0.9997	1.0000	0.9998	0.9931	6006
RAT (*class 1*)	1.0000	0.9524	0.9756	0.9931	42
Macro Avg	0.9998	0.9762	0.9877		6048
Weighted Avg	0.9997	0.9997	0.9997		6048

The marker-free object detection model evaluation metrics (6048 samples: 6005 background, 43 rat) trained with FOMO MobileNetV2 network.

## Data Availability

All the codes including the marker-based and marker-free methods for the treadmill have been uploaded to GitHub and are publicly available: https://github.com/ghli001/Real-Time-Vision-Based-Adaptive-Follow-Intelligent-Treadmill (accessed on 1 July 2025).
